# Intraoperative Anesthetic Management of Patients with Chronic Obstructive Pulmonary Disease to Decrease the Risk of Postoperative Pulmonary Complications after Abdominal Surgery

**DOI:** 10.3390/jcm9010150

**Published:** 2020-01-06

**Authors:** Sukhee Park, Eun Jung Oh, Sangbin Han, Beomsu Shin, Sun Hye Shin, Yunjoo Im, Yong Hoon Son, Hye Yun Park

**Affiliations:** 1 Department of Anesthesiology and Pain Medicine, International St. Mary’s Hospital, Catholic Kwandong University School of Medicine, Incheon 22711, Korea; appealex@gmail.com; 2 Department of Anesthesiology and Pain Medicine, Samsung Medical Center, Sungkyunkwan University School of Medicine, Seoul 06351, Korea; angy02@naver.com (E.J.O.); syhgood.son@samsung.com (Y.H.S.); 3 Department of Anesthesiology and Pain Medicine, Kangwon National University Hospital, Chuncheon 24341, Korea; 4 Department of Medicine, Yonsei University Wonju College of Medicine, Wonju 26426, Korea; bsshin83@gmail.com; 5 Division of Pulmonary and Critical Care Medicine, Department of Medicine, Samsung Medical Center, Sungkyunkwan University School of Medicine, Seoul 06351, Korea; fresh.shin@samsung.com (S.H.S.); yunjoo.im@samsung.com (Y.I.)

**Keywords:** airflow obstruction, chronic obstructive pulmonary disease, neuromuscular blocking reversal agent, postoperative pulmonary complications, protective lung ventilation

## Abstract

Patients with chronic obstructive pulmonary disease (COPD) exhibit airflow limitation and suboptimal lung function, and they are at high risk of developing postoperative pulmonary complications (PPCs). We aimed to determine the factors that would decrease PPC risk in patients with COPD. We retrospectively analyzed 419 patients with COPD who were registered in our institutional PPC database and had undergone an abdominal surgery under general anesthesia. PPCs comprised respiratory failure, pleural effusion, atelectasis, respiratory infection, and bronchospasm; the presence or type of PPC was diagnosed by respiratory physicians and recorded in the database before this study. Binary logistic regression was used for statistical analysis. Of the 419 patients, 121 patients (28.8%) experienced 200 PPCs. Multivariable analysis showed three modifiable anesthetic factors that could decrease PPC risk: low tidal volume ventilation, restricted fluid infusion, and sugammadex-induced neuromuscular blockade reversal. We found that the 90-day mortality risk was significantly greater in patients with PPC than in those without PPC (5.8% vs. 1.3%; *p* = 0.016). Therefore, PPC risk in patients with COPD can be decreased if low tidal volume ventilation, restricted fluid infusion, and sugammadex-induced reversal during abdominal surgery are efficiently managed, as these factors result in decreased postoperative mortality.

## 1. Introduction

Chronic obstructive pulmonary disease (COPD) is characterized by airflow limitation due to the narrowing of small airways and destruction of the lung parenchyma [1]. Suboptimal baseline lung function contributes to an increased risk of postoperative pulmonary complications (PPCs) in patients with COPD [1,2]. Even mild PPCs such as atelectasis or pleural effusion can worsen the clinical course and increase the risk of prolonged hospital stay; therefore, upon intensive care unit admission, as well as hospital readmission [3,4,5], anesthesiologists use various strategies—mechanical ventilation [6,7,8], fluid therapy [9], and neuromuscular blockade and its reversal [10,11]—to prevent PPCs. We hypothesized that there is a possibility of reducing PPCs using these strategies based on clinical evidence from patients who did not have COPD and who responded differently from patients with COPD due to the difference in lung physiology. To the best of our knowledge, so far, no study has evaluated the anesthetic factors that can prevent PPCs in patients with COPD; thus, we aimed to determine the intraoperative anesthetic factors that could modify PPC risk in patients with COPD undergoing abdominal surgery, in whom PPCs are commonly observed [12,13].

## 2. Materials and Methods

### 2.1. Subjects and Data Sources

**Figure 1:**
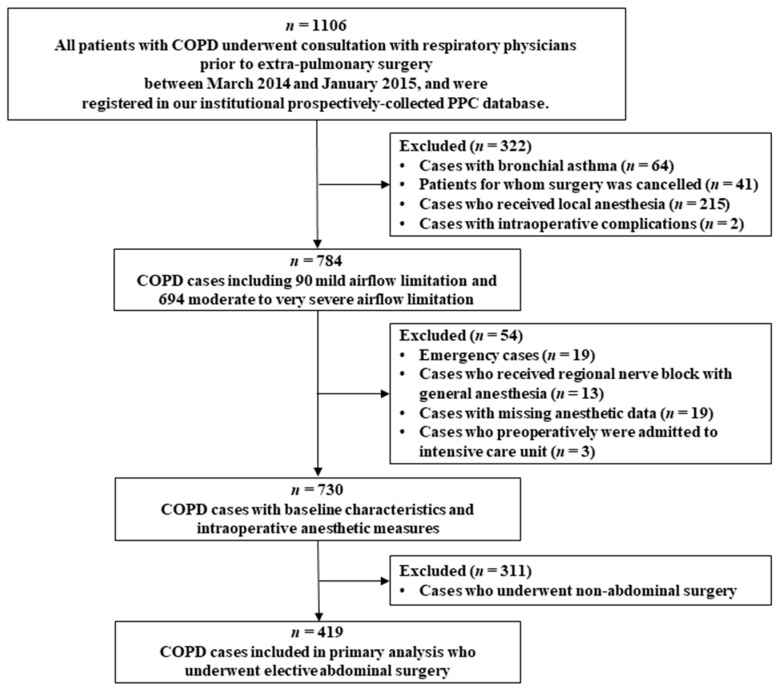
Consolidated standards of reporting trials diagram.

We initially screened 1106 patients with COPD who underwent preoperative consultations with respiratory physicians and were registered in our institutional, prospectively collected PPC database between March 2014 and January 2015 [14]. We excluded 64 patients with bronchial asthma, determined by Shin SH and Im Y based on the patients’ medical history with further confirmation by Park HY. Thereafter, we further excluded 623 patients owing to the reasons described in Figure 1. Finally, 419 patients with COPD who had undergone an elective abdominal surgery (upper abdominal surgery, *n* = 177; lower abdominal surgery, *n* = 132; and perineal surgery, *n* = 110) under isolated general anesthesia were included in this study. Upper abdominal surgery included pancreatectomy, gastrectomy, hepatectomy, cholecystectomy, small bowel resection, and abdominal aortic surgery. Lower abdominal surgery included colectomy, adrenalectomy, nephrectomy, and cystectomy. Perineal surgery included prostatectomy, endourological surgery, ureterostomy, ureteroureterostomy, oophorectomy, salpingo-oophorectomy, salpingectomy, hysterectomy, and uterine myomectomy. All patients underwent a lung expansion maneuver with incentive spirometry during the preoperative and postoperative periods [15]. Deep inspiration, active coughing, and sputum expectoration were encouraged during the postoperative period.

All preoperative and postoperative data (including the presence or type of PPCs) were already collected by respiratory physicians in the aforementioned PPC database before the start of this study. PPCs were defined as a composite of respiratory failure, pleural effusion, atelectasis, respiratory infection, pneumothorax, and bronchospasm within seven days postoperatively based on previously published articles [13,16]. In particular, bronchiectasis was assessed by reviewing chest radiographs or high-resolution chest computed tomography scans [17]. Bronchodilator use was defined as the use of an inhaled short- or long-acting bronchodilator during the perioperative period. The Assess Respiratory Risk in Surgical Patients in Catalonia (ARISCAT) score was calculated based on age, blood oxygen saturation, recent respiratory infection, anemia, surgical incision, and surgical duration [16].

Intraoperative anesthetic variables collected for this study from electronic medical records included intubation difficulty, anesthetic agent use, mechanical ventilation parameters, hemodynamics, fluid therapy use, blood loss, core temperature, airway humidification, vasoactive drug use, and neuromuscular blockade and its reversal. Furthermore, postoperative outcome variables collected from electronic medical records included prolonged mechanical ventilation >24 h, reintubation, length of hospital stay, and postoperative 30- or 90-day mortality. Our institutional review board approved this retrospective study (SMC 2018-11-092, Chairperson Prof. Lee Suk-Koo) and waived the requirement for written informed consent.

### 2.2. Degree of Airflow Limitation

Preoperative spirometry was performed using a Vmax 22 apparatus (SensorMedics, Yorba Linda, CA, USA) according to the American Thoracic Society/European Respiratory Society criteria [18]. Absolute values of forced expiratory volume in 1 s (FEV_1_) and forced vital capacity (FVC) were obtained, and percentages of the predicted values for FEV_1_ and FVC were calculated using a reference equation obtained in a representative Korean sample [19]. The patients were considered to have COPD if they had a FEV_1_:FVC ratio of <0.70 [20,21]. The airflow limitation degree was classified based on the recommendation of the Global Initiative for Chronic Obstructive Lung Disease; mild, moderate, severe, and very severe airflow limitations were defined as FEV_1_ ≥ 80%, 50%–79%, 30%–49%, and <30% of predicted values, respectively [1]. The patients were considered to have combined restrictive lung disease if they had an FVC of <80% of the predicted value [21].

### 2.3. Statistical Analysis

The primary outcome was PPC. The association between the anesthetic variables and PPC risk was analyzed using binary logistic regression, and the results were described as odds ratio (OR) and 95% confidence interval (CI). Stepwise backward selection was used for all the analyzed variables to generate a final multivariable model. The cutoff value for low tidal volume mechanical ventilation was set at 8 mL/kg of the ideal body weight [6,22]. The ARISCAT score was separately included in another multivariable model representing all the analyzed preoperative variables owing to overlap with other variables and concerns regarding multicollinearity. Exploratory analyses were performed to evaluate the effect of each protective anesthetic factor identified in this study on PPC risk within various high- or low-risk subgroups stratified according to preoperative risk factors identified in this study. We further evaluated the association between the protective anesthetic factors and PPC risk according to airway flow limitation degree. The secondary outcomes were prolonged postoperative mechanical ventilation, reintubation, length of postoperative hospital stay, and postoperative 90-day mortality. Continuous variables were summarized as median (25th percentile–75th percentile) and compared using *t*-test or Wilcoxon rank-sum test. Categorical variables were presented as frequency (percentage) and compared using chi-square test or Fisher’s exact test. All reported p values were two-sided, and *p* < 0.05 was considered statistically significant. Analyses were performed using SPSS 25.0 (IBM Corp., Chicago, IL, USA) or R 3.5.1 (R Development Core Team, Vienna, Austria; http://www.R-project.org).

## 3. Results

Of the 419 patients, 121 patients (28.8%) experienced 200 PCCs, with 54 patients (12.9%) experiencing multiple PPCs. The most common PPC was pleural effusion (*n* = 85, 20.2%), followed by atelectasis (*n* = 63, 15.0%), respiratory failure (*n* = 28, 6.7%), respiratory infection (*n* = 16, 3.8%), and bronchospasm (*n* = 8, 1.9%). None of the patients developed pneumothorax. Data on the first onset of PPC per patient were collected. The number of patients who initially developed PPC on postoperative days 0, 1, 2, 3, 4, and 5 was 38 (31.4%), 24 (19.8%), 49 (40.5%), 6 (5.0%), 2 (1.7%), and 2 (1.7%) patients, respectively.

The baseline characteristics of the 419 patients are described in Table 1. During the perioperative period, 103 patients (25%) underwent bronchodilator treatment. Among these 103 patients, 24 patients were treated with maintenance long-acting bronchodilators: 10 patients with long-acting muscarinic antagonist (LAMA), 7 patients with LAMA and long-acting beta 2-agonist (LABA) ± inhaled corticosteroid (ICS), and 7 patients with LABA ± ICS. The remaining 79 patients were treated with short-acting bronchodilators during the perioperative period: 47 patients with short-acting muscarinic antagonist (SAMA) and short-acting beta 2-agonist (SABA), 30 patients with SAMA, and 2 patients with SABA.

Neuromuscular blockade reversal was performed using a nondepolarizing agent (pyridostigmine or neostigmine) in 360 patients (81.4%), whereas sugammadex was used for reversal in 59 patients (14.1%). Low tidal volume ventilation was applied in 190 patients (45.3%): the applied tidal volume was 7.4 (6.8–7.7) mL/kg in patients with low tidal volume and 8.8 (8.4–9.3) mL/kg in those with conventional high tidal volume. Positive end-expiratory pressure (PEEP) ≥5 cmH2O was applied in 46 patients (11.0%): the applied PEEP was 2 (2–2) cmH2O in patients with <5 cmH2O PEEP and 5 (5–6) cmH2O in those with ≥5 cmH2O PEEP. Crystalloids were infused at a rate of 5.8 (4.4–7.1) mL/kg/h, and 81 patients (19.3%) underwent crystalloid infusion at a rate of < 4.0 mL/kg/h. The ARISCAT score was 41 (41–49) in patients with PPCs and 34 (19–41) in those without PPCs (*p* < 0.001).

Regarding the intraoperative variables (Table 2), the following factors were significantly associated with PPC risk (*p* < 0.05): neuromuscular blocking agent use, dynamic compliance, fluid infusion, red blood cell transfusion, blood loss, vasoactive drug use, sugammadex use, and prolonged general anesthesia. In contrast, PEEP was not significantly associated with PPC risk (*p =* 0.605).

### 3.1. Multivariable Analysis

As shown in Table 3, the results of the multivariable analysis demonstrated that old age >70 years (OR = 1.86 (1.10–3.15); *p* = 0.022), preoperative bronchiectasis (OR = 2.27 (1.10–4.68); *p* = 0.026), upper abdominal surgery [vs. perineal surgery, OR = 7.43 (3.02–18.29); *p <* 0.001], or lower abdominal surgery (vs. perineal surgery, OR = 3.40 (1.35–8.57); *p* = 0.009), and prolonged general anesthesia >3 h (OR = 2.75 (1.55–4.88); *p* = 0.001) were independent risk factors for PPCs. Regarding modifiable intraoperative anesthetic variables, low tidal volume ventilation (OR = 0.50 (0.29–0.85); *p* = 0.010), restricted crystalloid infusion (OR = 1.13 (1.03–1.25); *p* = 0.012), and sugammadex-induced neuromuscular blockade reversal (OR = 0.27 (0.11–0.69); *p* = 0.006) were identified as independent protective factors that could prevent PPCs. The type of neuromuscular blocking agent lost its significance in multivariable analysis after adjustment for sugammadex use and other variables.

The results of the multivariable analysis, with the ARISCAT score representing all the analyzed preoperative variables, confirmed the significance of the three protective anesthetic factors: low tidal volume ventilation (OR = 1.72 (1.02–2.91); *p* = 0.044), restricted crystalloid infusion (OR = 1.12 (1.01–1.23); *p* = 0.033), and sugammadex-induced neuromuscular blockade reversal (OR = 0.30 (0.12–0.73); *p* = 0.009). The ARISCAT score was also identified as a significant prognostic factor predicting PPC risk in patients with COPD (*p* < 0.001).

### 3.2. Respective Importance of Each Protective Factor on Each PPC

The effect of each identified protective anesthetic factor on each PPC is described in Supplementary Tables S1–S3. Tidal volume did not show a dominant association with any single PPC. The amount of crystalloid infusion was significantly associated with respiratory failure and pleural effusion. Sugammadex-induced neuromuscular blockade reversal was significantly associated with respiratory failure and pleural effusion while being associated with atelectasis with a marginal significance (*p* = 0.056).

### 3.3. Respective Importance of Each Protective Factor on PPC Risk According to the Existing Risk Factors

**Figure 2:**
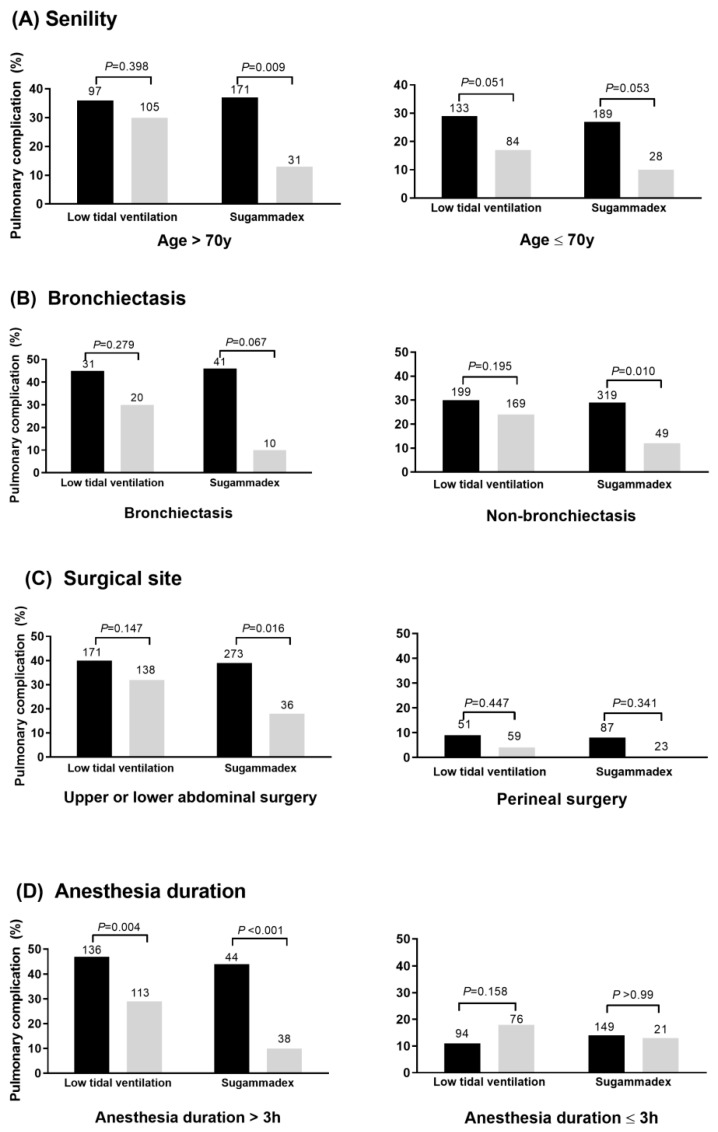
Respective impact of low tidal volume ventilation and sugammadex use in high- and low-risk subgroups stratified by (**A**) age, (**B**) bronchiectasis, (**C**) surgical site, and (**D**) anesthesia duration. The gray bar indicates the patients with low tidal volume ventilation or sugammadex use. The number above the bar indicates the total number of patients within the subgroup.

As shown in Figure 2, PPC risk was significantly decreased in relation to sugammadex use within the high-risk subgroups of patients with old age (>70 years), bronchiectasis, upper or lower abdominal surgery, and prolonged general anesthesia (>3 h). Fluid infusion rate was significantly higher in patients with PPC than in those without PPC within the high-risk subgroups of patients with old age (6.2 (5.0–8.4) vs. 5.6 (4.3–6.8); *p* = 0.005), upper or lower abdominal surgery (6.4 (4.9–7.7) vs. 5.9 (4.7–7.4); *p* = 0.018), and prolonged general anesthesia (6.0 (4.9–7.3) vs. 6.4 (5.1–7.5); *p* = 0.036). Although there was a consistent trend toward a lower PPC risk with low tidal volume ventilation within the various subgroups, low tidal volume ventilation was particularly effective in patients with >3 h anesthesia (*p* = 0.004). In the low-risk subgroup of patients with perineal operation or with <3 h anesthesia, the effect of low tidal volume ventilation and sugammadex use was limited. 

**Figure 3:**
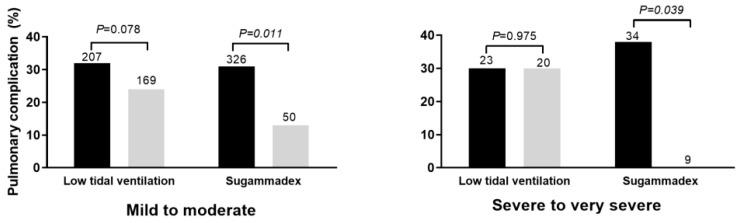
Respective impact of low tidal volume ventilation and sugammadex use according to airflow limitation degree. The gray bar indicates the patients with low tidal volume ventilation or sugammadex use. The number above the bar indicates the total number of patients within the subgroup.

As shown in Figure 3, PPC risk was significantly decreased in relation to sugammadex use, irrespective of airflow limitation degree. In contrast, fluid infusion (*p* < 0.001) was significantly associated with PPC risk only within the subgroup of patients with mild to moderate airflow limitation (6.4 (5.0–7.4) vs. 5.5 (4.2–6.6); *p* < 0.001), whereas no such significant association was observed within the subgroup of patients with severe to very severe airflow limitation (*p* = 0.694). Tidal volume showed a marginal significant association with PPC risk within the subgroup of patients with mild to moderate airflow limitation (*p* = 0.078), whereas no such association was observed within the subgroup of patients with severe to very severe airflow limitation (*p* = 0.975).

### 3.4. Cumulative Effect of Protective Anesthetic Interventions

**Figure 4:**
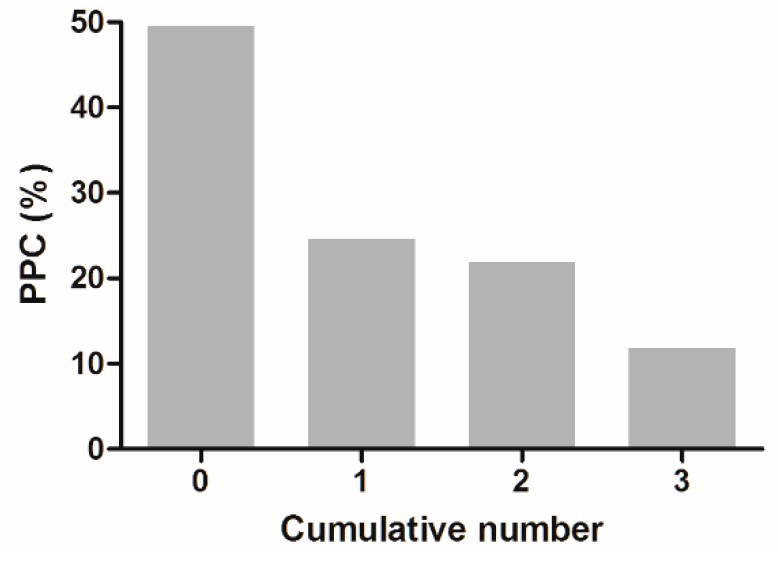
The incidence of postoperative pulmonary complications (PPCs) according to the number of cumulative protective anesthetic interventions.

As shown in Figure 4, the incidence of PPC was decreased in relation to the use of a greater number of interventions among the three protective anesthetic factors: 49.5% with none, 24.6% with one, 21.9% with two, and 11.8% with three. This finding suggested that the multimodal anesthetic strategy is advantageous for intraoperative lung protection and PPC prevention compared with the use of one dominant protective factor.

### 3.5. Association between PPC and Postoperative Clinical Courses

As shown in Table 4, the length of the postoperative hospital stay was insignificantly different between patients with and without PPCs (187.4 (161.8–251.7) h vs. 159.4 (61.1–203.8) h; *p* = 0.071). The risk of 90-day mortality was significantly greater in patients with PPCs than in those without PPCs (5.8% vs. 1.3%; *p* = 0.016).

## 4. Discussion

The present study determined the intraoperative anesthetic factors associated with PPCs in patients with COPD; we demonstrated that certain anesthetic strategies can modify PPC risk in this patient population. Our study cohort was derived from a prospectively collected PPC database of surgical patients who underwent preoperative consultations with respiratory physicians. Thus, data on the absence or presence of PPCs had already been collected in the database before the study started [14]. All the analyzed patients had nonasthmatic COPD. We found that PPC risk was significantly decreased with the use of low tidal volume ventilation, restricted crystalloid infusion, and sugammadex-induced neuromuscular blockade reversal. In particular, sugammadex use was dominantly associated with decreased respiratory failure, pleural effusion, and atelectasis; furthermore, the benefits of sugammadex were consistently observed in various high-risk subgroups of old age, bronchiectasis, upper or lower abdominal surgery, and prolonged general anesthesia as well as in patients with various airflow limitation degrees (mild to very severe). Although sugammadex showed a dominant effect, we found that a multimodal strategy, instead of a single intervention, is more effective for preventing PPCs. The ARISCAT score was found to show predictability even in patients with COPD, which is consistent with the findings of previous research [16]. The occurrence of PPCs was significantly associated with increased postoperative 90-day mortality. Thus, our findings should be incorporated into real clinical practice to prevent PPCs and to improve survival in patients with COPD. Interestingly, PEEP and driving pressure did not show any significant effect, which is in contrast to the findings of previous studies [8,23,24]. The insignificance suggests different responses to PEEP and driving pressure in patients with COPD, which may be due to different lung physiology.

The significant protective effect of low tidal volume ventilation in patients with COPD can be explained by the fact that the main pathophysiology of COPD is lung hyperinflation, which results from elastic recoil loss due to lung parenchyma destruction (so-called static hyperinflation) as well as airflow limitation due to small airway destruction (so-called dynamic hyperinflation) [25,26]. Lung hyperinflation sets the lung relaxation volume to a higher level than that in healthy subjects; thus, the end-expiratory lung volume and residual volume increase, whereas the inspiratory reserve volume decreases [27], resulting in a reduced capacity to further expand the tidal volume. Thus, the lungs may be more vulnerable to high tidal volume with an increased risk of barotrauma.

In contrast to the findings of recent studies on patients who did not have COPD, PEEP [28] and driving pressure [8,29] were not significantly associated with PPC risk in the present study. These conflicting results can be also explained by lung hyperinflation, which leads to intrinsic PEEP in patients with COPD. This intrinsic PEEP is known to reach 6–9 cmH_2_O in these patients [27,30]; thus, the actual PEEP in the alveolar unit is higher than the PEEP generated by the ventilator. It can be deduced that the actual PEEP (>8 cmH2O) was sufficient to prevent PPCs such as atelectasis even in patients with low PEEP [2 (2–2) cmH2O]. In the same manner, it can be deduced that the driving pressure (plateau pressure minus PEEP) was overestimated because we considered the PEEP generated by the ventilator instead of the actual PEEP in the alveolar unit. Because the driving pressure of the 419 patients was 9 (8–11) cmH_2_O, the actual driving pressure in the alveolar unit might have been lower than 14–18 cmH_2_O, which is thought be a safe range for preventing lung injury [29], even in patients with the highest quartile driving pressure.

The amount of infused crystalloids was positively correlated with PPC risk, which is in agreement with the findings of previous studies on patients who did not have COPD [9,31,32]: PPC risk increases by approximately 10% with every 1 mL/kg/h increase in fluid infusion. In particular, fluid infusion is dominantly associated with respiratory failure and pleural effusion. Perioperative fluid overload impairs the endothelial integrity and increases vascular permeability, thereby resulting in tissue inflammation and edema in various organs, including the lungs [31,32]. The lungs of patients with COPD may be more vulnerable to fluid overload owing to the already existing small airway inflammation and lung parenchyma destruction. Because the optimal amount of fluid infusion is dynamic, differing by the hemodynamics and the ongoing circulating volume changes [33], goal-directed fluid management using a minimum effective dose under the guidance of dynamic respiration-related parameters such as pleth variability index, pulse pressure variation, or stroke volume variation is highly recommended for the management of patients with COPD [34].

In this study, sugammadex consistently showed strong protective effects in various subgroups. Sugammadex immediately induces a solid and persistent reversal from neuromuscular blockade by encapsulating rocuronium or vecuronium molecules [35]. Recent studies have consistently reported that sugammadex prevents PPCs by avoiding the residual neuromuscular blockade, which is known to be common with conventional nondepolarizing agents [10,11,36]. Residual neuromuscular blockade interrupts the restoration of the normal activities of the respiratory muscles such as re-expansion of the atelectasis, effective cough for secretion removal, and coordination of the pharyngeal and upper esophageal muscles. The effect of residual neuromuscular blockade may be more significant in patients with COPD owing to the following reasons. Lung hyperinflation leads to diaphragm shortening with disturbances in the muscle force–length relationship while causing reduced force-generating capacity [37]. In addition, elastic recoil loss results in high inspiratory load [38]. These pathological alterations in the lungs may decrease the capacity of the inspiratory muscles to initiate work during neuromuscular blockade reversal and potentially make patients more vulnerable to residual paralysis, thereby supporting the dominant effect of sugammadex observed in this study.

This study has several limitations. First, as this was a retrospective study, we could not exclude the possibility of bias from unobserved (unmeasured or unmeasurable) variables. For instance, data on some anesthetic variables such as neuromuscular blockade degree (e.g., train-of-four ratio), passive airway humidifier use, and intermittent lung recruitment maneuver were not reliably recorded and could not be analyzed. Second, there may be patients with COPD who did not undergo consultations with respiratory physicians and who were not registered in the PPC database; this may have led to selection bias. However, we believe that the selection bias was not significant because it was routine for anesthesiologists to screen the presence of COPD and request for a missing preoperative consultation with respiratory physicians for PPC risk evaluation and perioperative respiratory management. Third, COPD was defined based on the prebronchodilator pulmonary function test, and some of the patients could be determined to be normal in the postbronchodilator pulmonary function test. Nonetheless, we thought that patients with airflow limitation in the present study had a high probability of having COPD because we excluded asthmatic patients. In addition, our results are more applicable to the real clinical practice in which many surgical patients do not undergo the postbronchodilator pulmonary function test owing to cost and time concerns. Fourth, we analyzed dynamic lung compliance, instead of static lung compliance, although static lung compliance is a more reliable parameter for assessing the elastic property of the lung in COPD patients [39], because some of the study patients (*n* = 30) were ventilated under pressure control mode and static lung compliance could not be measured [40,41].

In the present study, 28.8% of the patients experienced PPCs, with 12.9% of them experiencing multiple PPCs. We found evidence that low tidal volume ventilation, restrictive fluid infusion, and sugammadex-induced neuromuscular blockade reversal modify PPC risk in patients with COPD. Our results demonstrated that a multimodal strategy using all three protective factors is more advantageous than using a single strategy, although sugammadex showed the most prominent impact. In addition, we found that the occurrence of PPCs was associated with postoperative short-term mortality. Thus, the findings of our study suggest that anesthesiologists should be careful when performing mechanical ventilation, fluid therapy, and neuromuscular reversal for patients with COPD undergoing abdominal surgery under general anesthesia.

## Figures and Tables

**Table 1:** Preoperative variables of the patients.

	Without PPCs (*n* = 298)	With PPCs (*n* = 121)	*p*
Patient factors			
Age > 70 years	135 (45.3)	67 (55.4)	0.067
Male sex	243 (81.5)	107 (88.4)	0.109
Body mass index (kg/m^2^)	23.7 (21.5–25.3)	23.3 (22.0–25.2)	0.774
Diabetes	79 (26.5)	33 (27.3)	0.903
Hypertension	152 (51.0)	73 (60.3)	0.085
Hemoglobin (g/dL)	13.3 (11.8–14.5)	13.3 (11.9–14.2)	0.674
Neutrophil-to-lymphocyte ratio	1.9 (1.4–2.8)	1.9 (1.4–2.6)	0.701
Albumin (g/dL)	4.3 (4.0–4.5)	4.2 (4.0–4.5)	0.449
Creatinine (mg/dL)	0.9 (0.8–1.1)	0.9 (0.8–1.0)	0.664
ASA class 3 or 4	9 (3.0)	7 (5.8)	0.258
Arrhythmia on electrocardiography	16 (5.4)	11 (9.1)	0.188
Malignancy	196 (65.8)	104 (86.0)	<0.001
Smoking history			0.101
Never smoker	108 (36.2)	31 (25.6)	
Previous smoker	142 (47.7)	65 (53.7)	
Current smoker	48 (16.1)	25 (20.7)	
Bronchiectasis	31 (10.4)	20 (16.5)	0.099
Airflow limitation degree			0.972
Mild	36 (12.1)	15 (12.4)	
Moderate	232 (77.9)	93 (76.9)	
Severe to very severe	30 (10.1)	13 (10.7)	
Combined restrictive lung disease	131 (44.0)	62 (51.2)	0.195
Perioperative bronchodilator use	66 (22.1)	37 (30.6)	0.080
Procedure factors			
Surgical site			<0.001
Upper abdomen	99 (33.1)	78 (64.5)	
Lower abdomen	96 (32.2)	36 (29.8)	
Perineal	103 (34.6)	7 (5.8)	
Surgical method			0.022
Nonlaparoscopic	189 (63.2)	91 (75.2)	
Laparoscopic	109 (36.6)	30 (24.8)	
ARISCAT score	34 (19–41)	41 (41–19)	<0.001

Data are presented as median (25th percentile–75th percentile) or frequency (percent). ARISCAT, The Assess Respiratory Risk in Surgical Patients in Catalonia; ASA, American Society of Anesthesiologists; PPC, postoperative pulmonary complication.

**Table 2:** Intraoperative variables of the patients.

Intraoperative Variables	Without PPCs (*n* = 298)	With PPCs *(n* = 121)	*p*
Intubation grade moderate to difficult	30 (10.1)	6 (5.0)	0.123
Anesthetic maintenance agent			0.083
Sevoflurane	183 (61.4)	81 (66.9)	
Isoflurane	15 (19.2)	12 (9.9)	
Desflurane	81 (27.2)	23 (19.0)	
Propofol	19 (6.4)	5 (4.1)	
Neuromuscular blocking agent			<0.001
Rocuronium	126 (42.3)	21 (17.4)	
Vecuronium	133 (44.6)	81 (66.9)	
Cisatracurium	39 (13.0)	19 (15.7)	
Sugammadex-induced neuromuscular blockade reversal	52 (17.4)	7 (5.8)	0.002
Mechanical ventilation parameters			
Tidal volume (mL/kg IBW)	8.1 (7.4–8.8)	8.3 (7.6–9.0)	0.142
Low tidal volume ventilation	143 (47.8)	47 (38.8)	0.089
Peak pressure (cmH_2_O)	15 (14–17)	15.0 (13–17)	0.081
Plateau pressure (cmH_2_O)	12 (10–14)	12.0 (10–13)	0.289
PEEP (cmH_2_O)	2 (2–3)	2 (2–3)	0.422
PEEP ≥5 cmH_2_O	31 (10.4)	15 (12.4)	0.605
Respiratory rate (bpm)	10 (9–12)	10 (9–11)	0.200
Driving pressure	9 (8–11)	9 (8–11)	0.430
Dynamic compliance	30.2 (26.2–34.2)	31.9 (28.2–35.7)	0.010
Active airway humidification	71 (23.8)	27 (22.3)	0.800
Fluid therapy			
Crystalloid infusion (mL/kg/h)	5.6 (4.2–6.8)	6.4 (4.9–7.6)	<0.001
Colloid infusion	72 (24.2)	54 (44.6)	<0.001
Red blood cell transfusion	20 (6.7)	17 (14.0)	0.022
Hemodynamic parameters			
Estimated blood loss (mL)	100 (50–200)	200 (100–450)	<0.001
MBP <60 cmH2O for >30 min	14 (4.7)	12 (9.9)	0.071
Continuous vasoactive drug use	9 (3.0)	11 (8.3)	0.034
Hypothermia	210 (70.5)	92 (76.0)	0.280
Opioid use for intraoperative pain control			0.468
None	52 (17.4)	18 (14.9)	
Fentanyl	40 (13.4)	15 (12.4)	
Hydromorphone	120 (40.3)	59 (48.8)	
Demerol	45 (15.1)	12 (9.9)	
Morphine	41 (13.8)	17 (14.0)	
Anesthesia time >3 h	151 (50.7)	97 (80.2)	<0.001
Discharge to intensive care unit	60 (20.1)	59 (48.8)	<0.001

Data are presented as median (25th percentile–75th percentile) or frequency (percent). IBW, ideal body weight; MBP, mean blood pressure; PEEP, positive end-expiratory pressure; PPC, postoperative pulmonary complication.

**Table 3:** Multivariable analysis.

	OR	*p*
Nonmodifiable factor		
Age > 70 years	1.86 (1.10–3.15)	0.022
Preoperative bronchiectasis	2.27 (1.10–4.68)	0.026
Surgical site (vs. perineal)		
Upper abdomen	7.43 (3.02–18.29)	<0.001
Lower abdomen	3.40 (1.35–8.57)	0.009
Arrhythmia on electrocardiography	2.38 (0.87–6.52)	0.092
Smoking history (vs. never smoker)		
Previous smoker	1.78 (0.99–3.17)	0.055
Current smoker	1.98 (0.94–4.15)	0.073
Anesthesia time > 3 h	2.75 (1.55–4.88)	0.001
Discharge to intensive care unit	1.71 (0.96–3.05)	0.066
Modifiable factor		
Low tidal volume ventilation	0.50 (0.29–0.85)	0.010
Crystalloid infusion (mL/kg/h)	1.13 (1.03–1.25)	0.012
Sugammadex-induced neuromuscular blockade reversal	0.27 (0.11–0.69)	0.006

Peak inspiratory pressure was not included in the stepwise selection process owing to the mathematical connection to driving pressure. Tidal volume and positive end-expiratory pressure were not included in the stepwise selection process owing to the concern of multicollinearity with low tidal volume ventilation and high PEEP ventilation, respectively.

**Table 4:** Association between postoperative pulmonary complications (PPCs) and clinical courses.

	Without PPCs (*n* = 298)	With PPCs *(n* = 121)	*p*
**Prolonged mechanical ventilation > 24 h**	1 (0.3)	3 (0.7)	0.075
Reintubation	0	1 (0.8)	0.289
Postoperative length of hospital stay (h)	159.4 (61.1–203.8)	187.4 (161.8–251.7)	0.071
30-day mortality	1 (0.3)	1 (0.8)	0.495
90-day mortality	4 (1.3)	7 (5.8)	0.016

Data are presented as median (25th percentile–75th percentile) or frequency (percent).

## Supplementary Materials

The following are available online at https://www.mdpi.com/2077-0383/9/1/150/s1, Table S1: Association of tidal volume with each pulmonary complication; Table S2: Association of fluid infusion amount and each pulmonary complication; Table S3: Association of sugammadex use and each pulmonary complication. The English in this document has been checked by at least two professional editors, both native speakers of English. For a certificate, please see supplementary file.
