# Supplementary material for: Intraoperative Anesthetic Management of Patients with Chronic Obstructive Pulmonary Disease to Decrease the Risk of Postoperative Pulmonary Complications after Abdominal Surgery
Source: J Clin Med. 2020 Jan 6;9(1):150. doi: 10.3390/jcm9010150 (PMC7019772; doi:10.3390/jcm9010150)
Supplement: Supplementary file 1 [file jcm-09-00150-s001.zip › jcm-690470-SI.docx]

**Supplementary Information**

**Supplementary Table S1.** Association of tidal volume with each pulmonary complication.

|  | **Respiratory failure** | **Pleural effusion** | **Atelectasis** | **Respiratory**  **infection** | **Bronchospasm** |
| --- | --- | --- | --- | --- | --- |
| Low tidal volume | 13 (6.8) | 34 (17.9) | 24 (12.6) | 4 (2.1) | 2 (1.1) |
| High tidal volume | 15 (6.6) | 51 (22.3) | 39 (17.0) | 12 (5.2) | 6 (2.6) |
| *P* | 1.000 | 0.275 | 0.210 | 0.096 | 0.302 |

Data are presented as frequency (percent).

**Supplementary Table S2.** Association of fluid infusion amount and each pulmonary complication.

|  | **Crystalloid infusion (mL/kg/h)** | ***P*** |
| --- | --- | --- |
| Respiratory failure |  | 0.017 |
| No | 5.7 (4.4–7.0) |  |
| Yes | 6.7 (4.8–9.4) |  |
| Pleural effusion |  | <0.001 |
| No | 5. 6 (4.3–6.8) |  |
| Yes | 6.6 (5.2–8.7) |  |
| Atelectasis |  | 0.146 |
| No | 5.7 (4.3–7.2) |  |
| Yes | 6.0 (4.7–7.1) |  |
| Respiratory infection |  | 0.122 |
| No | 5.7 (4.4–7.1) |  |
| Yes | 6.6 (4.7–9.4) |  |
| Bronchospasm |  | 0.737 |
| No | 5.7 (4.4–7.2) |  |
| Yes | 6.3 (4.4–6.9) |  |

Data are presented as median (25th percentile–75th percentile).

**Supplementary Table S3.** Association of sugammadex use and each pulmonary complication.

|  | **Respiratory failure** | **Pleural effusion** | **Atelectasis** | **Respiratory infection** | **Bronchospasm** |
| --- | --- | --- | --- | --- | --- |
| Non-sugammadex | 28 (7.8) | 79 (21.9) | 59 (16.4) | 15 (4.2) | 7 (1.9) |
| With sugammadex | 0 | 6 (10.2) | 4 (6.8) | 1 (1.7) | 1 (1.7) |
| *P* | 0.022 | 0.037 | 0.056 | 0.711 | 1.000 |

Data are presented as frequency (percent).
